# First year of the pandemic: Years of Life Lost due to COVID-19 in Serbia

**DOI:** 10.1093/eurpub/ckac131.053

**Published:** 2022-10-25

**Authors:** A Stevanović, J Todorović, N Rosić, G Bjelobrk, S Mandić-Rajčević, M Šantrić-Milićević, V Bjegović-Mikanović

**Affiliations:** 1 Institute of Social Medicine, Faculty of Medicine, University of Belgrade, Belgrade, Serbia; 2 School of Public Health and Health Management, Faculty of Medicine, University of Belgrade, Belgrade, Serbia; 3 City Institute of Public Health, Belgrade, Serbia; 4 Statistical Office of the Republic of Serbia, Belgrade, Serbia

## Abstract

**Background:**

The first case of COVID-19 in Serbia was reported on 6th March 2020. Since then, Serbia has registered several epidemic peaks, which have led to a considerable increase in premature mortality. Basic evaluation of COVID-19 premature mortality burden needs to include frequency of COVID-19 deaths among different age-groups.

**Methods:**

All-cause mortality data disaggregated by age and sex, population estimates and remaining life expectancy for different age-groups were acquired from the Statistical Office of the Republic of Serbia. Years of Life Lost (YLL) due to COVID-19 were calculated for the period from March to December 2020. European Standard Population was used for calculating age-standardized mortality rates. We acknowledge the support from the BoCO-19 - The Burden of Disease due to COVID-19 project coordinated/led by Robert Koch Institute and supported by the WHO Regional Office for Europe.

**Results:**

In 2020, there were 127,572 YLLs due to COVID-19, with 81,147 of YLLs (63.6%) attributable to men and 46,425 (36.4%) to women. Contribution of COVID-19 to the total all-cause YLL was also higher in men comparing to women: 11.39% and 7.80%, respectively. Three epidemic peaks were observed in 2020, together composing two thirds (65.6%) of total YLLs due to COVID-19. December was the month with the greatest burden, accounting for 45.8% of all YLLs due to COVID-19. Crude YLL rate for COVID-19 was 1849.1 per 100,000, or 1733.5 per 100,000 after standardization.

**Conclusions:**

Registered COVID-19 deaths accounted for one tenth of total YLLs in Serbia in 2020, with men contributing almost twice as much to that number compared to women. On average, 12.32 YLLs originated from each registered COVID-19 death. Further studies need to assess the impact of the COVID-19 epidemic on avoidable mortality trends in Serbia.

**Key messages:**

• COVID-19 deaths comprised one tenth of all-cause YLLs, with two-thirds of COVID-19 YLLs attributable to men.

• To reduce premature mortality burden, epidemic peaks need to be prevented.

